# A dietary intervention for chronic diabetic neuropathy pain: a randomized controlled pilot study

**DOI:** 10.1038/nutd.2015.8

**Published:** 2015-05-26

**Authors:** A E Bunner, C L Wells, J Gonzales, U Agarwal, E Bayat, N D Barnard

**Affiliations:** 1 Physicians Committee for Responsible Medicine, Washington, DC, USA; 2 California State University, East Bay, Student Health and Counseling Services, Hayward, CA, USA; 3 George Washington Medical Faculty Associates, Washington, DC, USA; 4 George Washington University School of Medicine, Washington, DC, USA

## Abstract

**Background:**

Diabetic neuropathy is a common and often debilitating condition for which available treatments are limited. Because a low-fat plant-based diet has been shown to improve glycemic control in individuals with type 2 diabetes, we hypothesized that such a diet would reduce painful symptoms of diabetic neuropathy.

**Methods:**

In this 20-week pilot study, individuals with type 2 diabetes and painful diabetic neuropathy were randomly assigned to two groups. The intervention group was asked to follow a low-fat, plant-based diet, with weekly classes for support in following the prescribed diet, and to take a vitamin B12 supplement. The control group was asked to take the same vitamin B12 supplement, but received no other intervention. At baseline, midpoint and 20 weeks, clinical, laboratory and questionnaire data were collected. Questionnaires included an analog ‘worst pain' scale, Michigan Neuropathy Screening Instrument, global impression scale, Short Form McGill Pain Questionnaire, Neuropathy Total Symptom Score, a weekly pain diary and Norfolk Quality of Life Questionnaire.

**Results:**

After 20 weeks, body weight change with the intervention was −6.4 kg (95% confidence interval (CI) −9.4 to −3.4, *P*<0.001) in an effect size analysis. Electrochemical skin conductance in the foot improved by an average of 12.4 microseimens (95% CI 1.2–23.6, *P*=0.03) with the intervention in an effect size analysis. The between-group difference in change in pain, as measured by the McGill pain questionnaire, was −8.2 points (95% CI −16.1 to −0.3, *P*=0.04). Michigan Neuropathy Screening Instrument questionnaire score change was −1.6 points (95% CI −3.0 to −0.2, *P*=0.03).

**Conclusions:**

Improvements were seen in some clinical and pain measures. This pilot study suggests the potential value of a plant-based diet intervention, including weekly support classes, for treating painful diabetic neuropathy.

## Background

Diabetic peripheral neuropathy occurs in up to 60% of individuals with type 2 diabetes^1^ and is associated with significant morbidity, including gait disturbances, amputations, anxiety, depression and reduced quality of life.^2, 3^ The condition manifests with damage to the terminal branches of peripheral nerves and usually first affects small fibers that are responsible for translating pain, light touch and temperature. As neuropathy progresses, large fibers responsible for reflexes and muscle tone are affected, leading to balance and gait problems. Most patients with diabetic peripheral neuropathy present with pain, numbness, or abnormal, spontaneous or induced sensations in the lower extremities. Pain occurs in 15–30% of cases.^4^

Plant-based dietary interventions typically improve glycemic control and other factors associated with type 2 diabetes and its complications. In a study of 99 type 2 diabetes patients comparing a low-fat plant-based diet with a more conventional diet, in an analysis limited to participants making no medication changes, HbA1c (percent hemoglobin A1c) fell by 1.2 points in the plant-based group, compared with 0.4 points in the control group.^5^ Glycemic control appears to have a key role in the risk of complications.^6^ In intervention trials using plant-based diets, improvements in glycemic control,^6^ blood lipid concentrations^7^ and blood pressure^8^ have been consistently observed,^6, 7, 8^ and diet acceptability is similar to that of other therapeutic diets.^9^

Two uncontrolled diet intervention studies showed promising results in individuals with diabetic neuropathy.^10, 11^ We therefore hypothesized that a plant-based dietary intervention can reduce diabetic neuropathy pain and conducted a randomized, controlled, pilot study to test this hypothesis. This study was intended to investigate the efficacy of this approach and the suitability of the overall method, permitting larger trials to follow. The study was not intended to elucidate the mechanisms by which a dietary intervention might lead to clinical changes or to separate which parts of the intervention might be responsible for any observed benefit.

## Materials and methods

### Participants and recruitment

Participants were recruited in the Washington, DC, area through local physicians and media outlets in two replications. Inclusion criteria were age 18–65 years, diagnosis of type 2 diabetes, and diagnosis or symptoms of painful diabetic neuropathy for at least 6 months. Exclusion criteria were vitamin B12 deficiency, alcohol consumption of more than two drinks per day, use of recreational drugs in the past 6 months, pregnancy, unstable medical or psychiatric illness, current adherence to a vegan diet and inability or unwillingness to participate in all components of the study. Participants were screened for B12 deficiency, which can cause neuropathy independent of diabetes, by a B12 blood level test and a serum methylmalonic acid^12^ test. Criteria for deficiency were a B12 level of <200 pg ml^−^^1^ or a B12 level of <400 pg ml^−^^1^ in combination with MMA level >270 nmol ml^−^^1^.^12^

A screening interview was conducted to determine eligibility. Participants were determined to have symptoms of painful diabetic neuropathy if they described in the screening interview painful sensations in the hands or feet, including tingling, burning or freezing. The study was approved by Ethical and Independent Review Services. All participants signed a consent form. The trial was registered at Clinicaltrials.gov under NCT01690962 and NCT01953757.

Thirty-five participants were assigned to the intervention or control groups by study staff using an allocation sequence generated from a random number table. For the first replication, allocation concealment was achieved using sealed envelopes. Participants in the second replication were stratified by Neuropathy Total Symptom Scores in blocks of two.^13^ Since those assignments were done simultaneously, allocation concealment was not required. It was not possible to blind participants and instructors to group assignment. However, examinations for the Michigan Neuropathy Screening Instrument^14^ and Neuropathy Impairment Score-Lower Limb^15^ were performed by an independent blinded clinician who was impartial to the study hypothesis.

### Study design and procedures

The intervention group was instructed to follow the intervention diet and attend weekly nutrition classes offering education and social support for 20 weeks. The study duration was chosen to be comparable to that of previous studies demonstrating clinically significant effects of plant-based diets on type 2 diabetes.^16^ The intervention diet omitted animal products, limited fat intake to 20–30 g day^−^^1^ and favored low-glycemic index foods. The diet focused on vegetables, fruits, grains and legumes. Example meals included oatmeal with raisins, pasta with marinara sauce, vegetable stir-fry with rice and lentil stew. In addition, participants were asked to take a provided daily tablet of 1000 mcg methylcobalamin (vitamin B12).

The control group was asked to take the same provided vitamin B12 supplement daily but was asked to make no major diet changes during the 20-week study period. The B12 supplement was intended to prevent B12 deficiency in intervention-group participants, should they elect to continue the prescribed diet long term, and also to provide a credible placebo for the control group.^16^ Assessments occurred at baseline, 10 weeks and 20 weeks.

Participants were asked to keep their diabetes medications constant when possible, but to follow the advice of their personal physicians regarding medication use (for example, in the case of hypoglycemia).

### Dependent variables

Dietary changes were assessed through the use of 2-day dietary records analyzed by a registered dietitian. Body weight was measured in light clothing and without shoes using a digital scale accurate to 0.1 kg. Blood pressure was measured 3 times, using a digital sphygmomanometer. The first measurement was disregarded, and the mean of the remaining two measurements was calculated. Blood glucose, HbA1c (percent hemoglobin A1c) and plasma lipid concentrations were measured by standard methods at Quest Diagnostics (Baltimore, MD, USA).

Sensory perception was assessed by monofilament sensation, vibration perception and ankle reflex as part of the Michigan Neuropathy Screening Instrument physical assessment (MNSI-PA, this instrument also includes a questionnaire filled out by the patient, as noted below), and pin-prick and touch pressure were assessed as part of the Neuropathy Impairment Score-Lower Limb.^14, 15^ Electrochemical skin conductance was measured by a Sudoscan device, which evaluates each foot and each hand.^17, 18^ Electrochemical skin conductance is a measure of sudomotor nerve function, and is correlated with pain.^19^

Our primary outcomes were pain and sensory symptoms as measured by visual analog ‘worst pain' scale, global impression scale,^20^ Short Form McGill Pain Questionnaire,^21, 22^ Michigan Neuropathy Screening Instrument questionnaire (MNSI-Q), Neuropathy Total Symptom Score, a weekly pain diary, and Norfolk Quality of Life Questionnaire.^23, 24^ The visual analog scale was a 10-cm line anchored by ‘No pain' and ‘Pain as bad as it could possibly be' on either end, and participants were asked to rate their worst pain in the preceding 2 weeks. The visual analog scale was completed with an interviewer, but all other questionnaires were completed independently.

The patient's global impression of change (PGIC) question assesses subjective pain improvement by asking participants to rate symptom change on a scale of 1–7 from ‘no change' to ‘a great deal better.^20^

Pain diaries have been used in diabetic neuropathy clinical trials to track pain over time.^25^ Participants were asked to provide weekly ratings for average pain, worst pain and night pain on an 11-point numerical scale.

Mood and depression were measured by the Beck Depression Inventory (BDI)^26^ and the Center for Epidemiological Studies Depression revised scale.^27^ The BDI measures symptom severity, while the Center for Epidemiological Studies Depression revised scale measures symptom frequency.

### Statistical analysis

Since little has been published on the effects of a plant-based diet on pain in diabetic neuropathy and no prior controlled studies using a plant-based diet were found, a power analysis could not be based on the previous research. Previous studies were conducted on pre-diabetes patients^10^ or in a short-term residential program with no control group.^11^ Therefore, we chose an exploratory approach that did not limit sample size and accepted all volunteers who met the participation criteria.

For pain scales, body weight and lipid concentrations (total cholesterol, low density lipoprotein-cholesterol, high density lipoprotein-cholesterol and triglycerides), baseline descriptive statistics were calculated. For normally distributed data, two-tailed Student's *T*-tests for independent samples were calculated for 20-week changes in the diet and control groups. Since primary outcomes were based on the 20-week study endpoint, statistical approaches that evaluate changes over time were not used. For non-normally distributed variables, Wilcoxon signed-rank tests were used. Chi-squared tests were used for categorical variables. An alpha of 0.05 was used for all statistical tests. Baseline values for key outcome variables were included as covariates in the main assessments of the effect of diet in the multivariate analysis of covariance.

For missing data in a reporting period, values from the previous period were brought forward. For body weight, drop-outs were considered to have returned to baseline weights.

## Results

### Recruitment

**Figure 1:**
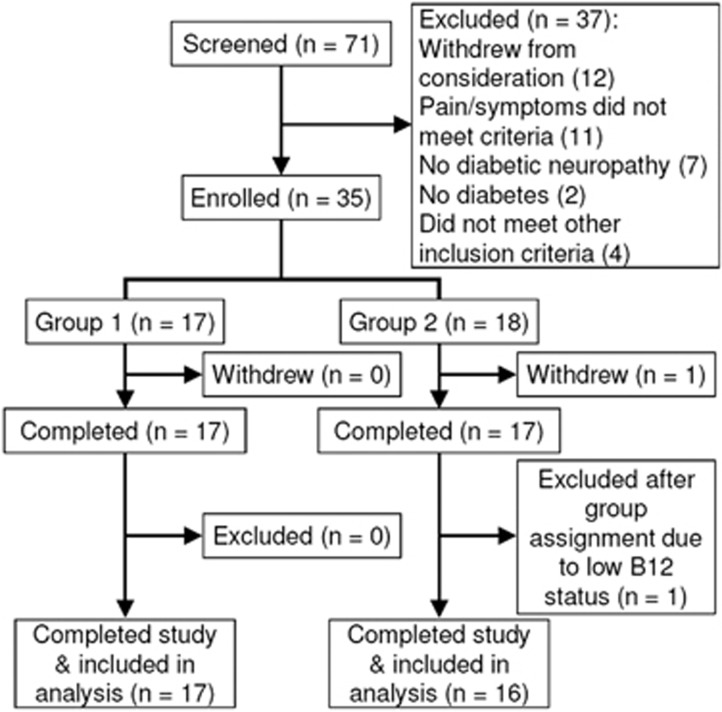
Recruitment and retention. Study completers attended the final assessment at 20 weeks. All but one participant were included in the analysis.

Seventy-one participants were interviewed and thirty-five were enrolled (Figure 1). Recruitment periods were November 2012 to January 2013 and October 2013 to January 2014. Among volunteers who were excluded or withdrew, compared with enrolled participants, fewer (4%) were Hispanic, more (64%) were black and fewer (31%) were white. One control-group participant was excluded from the data analysis on the basis of B12 deficiency. The participant group had a mean age of 57 years and was 56% women, 47% black and 15% Hispanic; 53% of participants had a college degree or a higher education (Table 1). There were no significant baseline differences between the two groups except for Neuropathy Impairment Score Lower Limb (NIS-LL), for which the intervention group had a higher score, indicating more severe neuropathy. At baseline, five intervention-group participants and five control-group participants were taking medications for diabetic neuropathy symptoms, including pregabalin, gabapentin, sertraline, duloxetine, bupropion and escitalopram. There were no significant study-related adverse effects. The study ended as per the protocol.

### Diet adherence

Two-day diet records conducted at midpoint and 20 weeks showed that 13 of 17 intervention-group participants avoided all animal products at the midpoint and endpoint assessments. Of those 13, 8 reported consuming a low-fat (25% kcal or less from fat) diet at both time points. An additional three of those thirteen reported consuming a low-fat diet at one of the two time points. An additional 2 of the 17 intervention-group participants were fully compliant with the low-fat guidelines at both assessments, but reported consuming at least modest amounts of animal products in one diet record, and two participants were non-compliant with the low-fat guidelines and the plant-based guidelines. No data on vitamin B12 supplement adherence were collected.

### Changes on clinical measurements

Body weight declined by a mean of 7.0 kg over 20 weeks in the intervention group, as compared with 0.6 kg in the control group (*P*<0.001, Table 2). Mean HbA1c declined in the intervention group by 0.8 percentage point over 20 weeks, but remained unchanged in the control group (*P*=0.07). Despite the request that participants not change medications, several did have medication adjustments, typically due to hypoglycemia. Glucose-lowering medications were reduced for 10 intervention-group participants and increased for two by their primary physicians. In the control group, glucose-lowering medications were reduced for one participant and increased for two.

Total cholesterol declined 12.1 mg dl^−^^1^ in the intervention group and 2.2 mg dl^−^^1^ in the control group (*P*=0.20). Low density lipoprotein cholesterol declined 7.8 mg dl^−^^1^ in the intervention group, but increased by 0.4 mg dl^−^^1^ in the control group (*P*=0.35). Compared with the control group, more intervention group participants reduced lipid-lowering medications (four vs zero) and fewer increased lipid-lowering medications (one vs three).

Systolic and diastolic blood pressure fell in both groups, without a significant between-group difference. During the 20 weeks, blood pressure medications were changed for six intervention-group participants (two reduced, four increased) and one control-group participant (zero reduced, one increased).

An analysis limited to study completers found similar results (Supplementary Table S1).

### Changes in perceived pain and neuropathy symptoms

Average foot conductance as measured by a Sudoscan device showed a decrease in sudomotor nerve function in the control group (−11.7 microseimens), but a non-significant improvement in the intervention group (0.7 microseimens, *P*=0.03). The decline in the control group was mostly due to a steep drop in measurements for two participants. A similar finding was observed in average hand conductance (*P*=0.14).

Pain, as measured by the Short Form McGill Pain Questionnaire, declined by 9.1 points in the intervention group over 20 weeks and by 0.9 points in the control group (*P*=0.04, Table 2). The largest decline was seen in the sensory subscore (Supplementary Table S2). Quality of life improved significantly within the intervention group but changes between group did not reach statistical significance. For the autonomic symptoms subscore of the quality of life questionnaire, the intervention group improved significantly, while the control group did not (*P*=0.05, Supplementary Table S3). Pain as measured by the Neuropathy Total Symptom Score and visual analog pain scale declined in both groups (*P*=0.70 and 0.39, respectively). The intervention group had significant improvements for seven of the ten items on the Neuropathic Pain Scale, but these differences were not significant between groups (Supplementary Table S4).

For the MNSI-PA, improvements were noted for both groups, but did not reach statistical significance for either group. For the MNSI-Q, the improvement for the intervention group was significantly greater than the improvement for the control group. For the Neuropathy Impairment Score-Lower Limb, the intervention group improved slightly, while the control group worsened, but neither result was statistically significant.

During the study, two intervention-group participants began taking medications for neuropathy, and two eliminated such medications. In the control group, one participant began and then stopped taking pregabalin. Limiting the analysis of symptom-related outcomes to participants who did not change symptom management medications did not change the results, except that changes in total Quality of Life score were no longer significant within the intervention group.

### Changes in mood

No significant changes were observed in the Beck Depression Inventory or the Center for Epidemiological Studies Depression revised scale over the 20 weeks.

## Discussion

In this 20-week diet intervention, body weight and body mass index decreased more in the intervention group, and HbA1c declined significantly only in the intervention group. Electrochemical skin conductance in the foot declined in the control group, but stayed essentially constant in the intervention group, suggesting that the intervention may have slowed or halted sudomotor nerve function decline. Significant improvements in pain were observed in the intervention group, as measured by the Quality of Life questionnaire, Short Form McGill Pain Questionnaire, MNSI-Q and change in pain question. Improvements in self-reported worst pain were observed in the control group, and improvements in Neuropathy Total Symptom Score were observed in both groups.

Our findings, using a design including a control group with a vitamin B12 supplement serving as a placebo control, are consistent with those of previous, smaller studies showing improvements in neuropathy pain and symptoms with a dietary intervention. Crane and Sample^11^ enrolled 21 participants with type 2 diabetes and painful neuropathy in a residential lifestyle intervention program, using a reduced-calorie, low-fat, plant-based diet with exercise for 25 days. Complete remission of burning pain and an improved sense of touch was reported by 81% of participants, while the remaining participants reported partial symptomatic relief. Other favorable changes included weight loss, decreased blood lipid concentrations and reduced need for medications for blood pressure and blood glucose control. That study was shorter in duration than the present study, and had a higher-intensity intervention, including a residential program with exercise. Since nerve damage occurs over the course of years, a longer study might be expected to show greater improvements in pain and neuropathy symptoms.

Smith *et al.*^10^ studied 32 individuals without diabetes but with impaired glucose tolerance and peripheral neuropathy. Participants were counseled on diet and exercise during the 1-year study. Improvements in glucose tolerance, body weight and cholesterol levels were accompanied by significant increases in intraepidermal nerve fiber density and foot sweat volume. Non-significant improvements in pain were observed. The intervention in the present study was more intense, with weekly nutrition education meetings, but had a shorter duration.

The mechanism(s) by which the low-fat plant-based diet improves neuropathy pain may involve improved insulin sensitivity, leading to better glucose control.^28^ In addition, diabetic neuropathy is associated with hypertension, dyslipidemia and obesity, all of which can be ameliorated with a plant-based diet.^8, 29, 30^

Adherence to the plant-based aspect of the diet was high, but adherence the low-fat requirement of the diet was less so. A previous review has shown that group support and frequent monitoring of dietary adherence improve adherence to reduced-fat diets, so those factors were included in this study.^30^

Medication changes in the intervention group may have obscured some improvements in glucose control, blood lipids and blood pressure. Most of the intervention-group participants who altered diabetes medications did so because of hypoglycemia. It is possible that some changes in pain and other symptoms were in part related to changes in symptom management medication that occurred during the 20 weeks.

It is noteworthy that the control group reported significant improvements in self-reported worst pain (visual analog pain) and symptoms as measured by the Neuropathy Total Symptom Score. The magnitude of the improvement suggests that the B12 supplement, intended to serve as a placebo, may have had real effects in both groups.^16^ Although all participants included in the data analysis had acceptable B12 and MMA levels at baseline, it is conceivable that some participants in both groups had increased B12 activity as a result of the supplement. This may have caused some of the observed improvements in the control group, particularly since 72% of the control participants (13 of 18), were metformin-treated, and B12 deficiency is more common in that population.^31^ In addition, the control group had a higher baseline mean body weight and a greater proportion of black or Hispanic participants (15 of 18 participants, compared with 6 of 17 in the intervention group). Among diabetes patients, blacks have a higher risk of diabetic neuropathy.^32^ We also note that, as used in the present study, the visual analog pain rated only ‘worst pain' during the preceding 2-week period, and did not assess typical or ‘average' pain.

This pilot study has several strengths. It used a randomized design, a control group, and an adequate time frame for the observation of clinical changes. It was conducted with community volunteers; therefore, the results are readily translatable to applications outside the research setting. A comparable previous study^11^ was done as part of a residential program, in which food was provided and exercise was mandatory. Those authors saw dramatic improvements in a short time, but they did not demonstrate that their intervention was sustainable or their results generalizable.

The study also has weaknesses. Because the study was designed to assess the efficacy of the intervention on neuropathy pain and related signs and not to determine which part of the intervention was responsible for observed clinical changes, the effect of the weekly classes cannot be separated from the effect of the diet. In addition, the effects of the plant-based diet cannot be differentiated from the effects of weight loss it may cause. Further studies are needed to identify the specific mechanisms by which the intervention may lead to physical and symptomatic improvements. The self-reported nature of pain is an intrinsic limitation of pain research. Although B12-deficient participants were excluded, we cannot be certain that the supplement, intended as a placebo, did not have real effects. Also, the definition of painful diabetic neuropathy as an inclusion criterion may have been insufficiently precise; future trials should ensure that diagnostic criteria adhere to current guidelines.

In conclusion, this pilot study suggests the potential of a dietary approach for treating diabetic neuropathy and provides findings that can be used to guide further studies. This was the first randomized controlled study of diet and diabetic nerve pain, and demonstrates that the effect of the diet is greater than a control treatment for some outcomes.

Nutrition studies are particularly important given that drugs used to manage blood glucose have significant side effects, and drugs used for diabetic nerve pain typically offer only limited benefit. Further studies might eliminate the confounding factor of vitamin B12 status by including vitamin B12 pretreatment or an alternative control, such as a subclinical dose of B12. A trial testing diet, exercise and vitamin B12 in combination might be a useful means of assessing the effects of a more intensive intervention in this otherwise intractable and debilitating chronic disease.

## Figures and Tables

**Table 1:** Baseline characteristics

*Variable*	*All particiants* (N=*34*)	*Intervention group* (N=*17*)	*Supplement group* (N=*17*)	P-value*
Women, *N*	19	11 (65%)	8 (47%)	0.56
College degree, *N*	18	10 (59%)	8 (47%)	0.88
Black race, *N*	16	5 (29%)	11 (65%)	0.09
Hispanic, *N*	5	1 (6%)	4 (24%)	0.34
Age, years	57 (6)^a^	57 (6)	58 (6)	0.48
Weight, kg	104 (23)	102 (23)	106 (24)	0.70
BMI, kg m^−^^2^	36 (6)	36 (6)	36 (7)	0.90
Diabetes duration, years	14 (10)	15 (11)	12 (10)	0.60
Total cholesterol, mg dl^−^^1^	172 (44)	171 (48)	173 (41)	0.89
Triglycerides, mg dl^−^^1^	152 (77)	151 (66)	153 (89)	0.95
HbA1c, %	7.9 (1.6)	8.0 (1.7)	7.8 (1.6)	0.78
HbA1c, mmol ml^−^^1^	63 (18)	64 (19)	62 (18)	0.78
Fasting glucose, mg dl^−^^1^	159 (66)	161 (74)	157 (60)	0.89
MNSI-PA	4.1 (2.3)	4.8 (2.4)	3.4 (2.2)	0.09
NIS-LL	8.9 (6.9)	11.3 (8.0)	6.6 (4.6)	0.05
Visual analog scale, cm	5.5 (2.6)	5.3 (2.7)	5.8 (2.4)	0.57
MPQ-T	22 (10)	23 (11)	21 (10)	0.65
QOL-T	29 (15)	28 (14)	30 (16)	0.74
NTSS-6	12 (5)	11 (5)	12 (5)	0.35
On metformin	24	12 (71%)	12 (71%)	0.99
On insulin	15	6 (35%)	9 (53%)	0.30
On other agents	17	10 (59%)	7 (41%)	0.31

Abbreviations: BMI, body mass index; MNSI-PA, Michigan Neuropathy Screening Instrument physical assessment; MPQ-T, Short form McGill Pain Questionnaire total; NIS-LL, Neuropathy Impairment Score Lower Limb; NTSS-6, Neuropathy Total Symptom Score; QOL-T, Norfolk diabetic neuropathy Quality of Life, total score.

a Scalar data are presented as mean (s.d.)

*P* values from Chi-squared tests or Student's *T*-tests.

**Table 2:** Intervention effects on clinical measures, intention-to-treat analysis

	*Intervention group*			*Supplement group*			*Effect size*	P-value*
	*Baseline*	*20 weeks*	*Change scores*	*Baseline*	*20 weeks*	*Change scores*	*Mean (95% CI)*	
	*Mean (s.d.)*	*Mean (s.d.)*	*Mean (s.d.)*	*Mean (s.d.)*	*Mean (s.d.)*	*Mean (s.d.)*		
Body weight (kg) (*N*=34)	102.5 (22.7)	95.5 (20.1)	−7.0 (5.0)^†^	105.6 (23.6)	105.1 (22.9)	−0.6 (3.5)	−6.4 (−9.4 to −3.4)	<0.001
BMI (*N*=34)	35.9 (6.0)	33.5 (5.7)	−2.4 (1.5)^†^	36.2 (7.1)	36.0 (7.1)	−0.2 (1.2)	−2.2 (−3.2 to −1.2)	<0.001
Total cholesterol (mg dl^−^^1^) (*N*=34)	171.2 (48.4)	159.1 (42.6)	−12.1 (37.8)	173.4 (41.4)	175.6 (34.0)	2.2 (25.6)	−14.4 (−36.9 to 8.2)	0.20
HDL (*N*=34)	52.1 (15.1)	46.8 (11.4)	−5.2 (10.1)^‡^	47.4 (18.9)	45.0 (16.9)	−2.4 (6.9)	−2.9 (−8.9 to 3.2)	0.34
LDL (*N*=34)	89.0 (38.3)	81.2 (34.2)	−7.8 (28.9)	95.4 (43.6)	95.8 (34.5)	0.4 (21.0)	−8.2 (−25.8 to 9.5)	0.35
Ratio (*N*=34)	3.4 (1.1)	3.5 (0.9)	0.1 (0.7)	4.0 (1.4)	4.3 (1.5)	0.3 (0.6)	−0.2 (−0.6 to 0.2)	0.36
Triglycerides (*N*=34)	151.0 (66.3)	155.7 (61.6)	4.7 (56.2)	152.6 (88.8)	174.5 (110.6)	21.9 (68.9)^a^	−17.2 (−61.2 to 26.8)	0.43
Log triglycerides (*N*=34)	2.14 (0.19)	2.16 (0.19)	0.01 (0.17)	2.12 (0.23)	2.17 (0.24)	0.05 (0.17)^a^	−0.04 (−0.16 to 0.08)	0.51
Fasting glucose (*N*=34)	160.6 (73.5)	134.6 (51.6)	−25.9 (65.0)	157.4 (60.5)	138.2 (51.3)	−19.2 (55.6)^a^	−6.8 (−49.0 to 35.5)	0.75
HbA1c, % (*N*=34)	8.0 (1.7)	7.2 (1.4)	−0.8 (1.2)^‡^	7.8 (1.6)	7.8 (1.4)	0.0 (0.9)	−0.7 (−1.5 to 0.1)	0.07
HbA1c, mmol ml^−^^1^ (*N*=34)	64 (19)	55 (15)	−9 (13)^‡^	62 (18)	62 (15)	0 (10)	−8 (−16 to 1)	0.07
Systolic BP (*N*=34)	139.3 (17.0)	127.9 (22.6)	−11.5 (22.5)^a^	144.4 (21.6)	140.1 (19.3)	-4.3 (15.0)	−7.2 (−20.6 to 6.2)	0.28
Diastolic BP (*N*=34)	81.5 (9.7)	75.8 (9.0)	−5.7 (9.4)^‡^	87.2 (14.5)	84.4 (10.4)	−2.8 (8.8)	−2.9 (−9.2 to 3.5)	0.36
Foot conductance, avg (*N*=20)^b^	64.5 (29.5)	65.2 (24.1)	0.7 (10.5)	73.4 (16.8)	61.7 (24.4)	−11.7 (13.2)^‡^	12.4 (1.2 to 23.6)	0.03
Hand conductance, avg (*N*=20)	53.2 (28.0)	55.7 (21.6)	2.5 (13.9)	67.6 (23.4)	61.2 (25.5)	−6.4 (11.7)	8.9 (−3.2 to 21.0)	0.14
MNSI-PA (*N*=34)	4.8 (2.4)	4.3 (2.3)	−0.5 (1.9)^a^	3.4 (2.2)	2.6 (1.8)	−0.8 (1.7)^a^	0.3 (−1.0 to 1.5)	0.67
NIS-LL (*N*=34)	11.3 (8.0)	8.7 (6.8)	−2.6 (7.3)	6.6 (4.6)	6.8 (5.3)	0.2 (4.9)	−2.8 (−7.1 to 1.6)	0.20
VAS (*N*=34)	5.3 (2.7)	4.0 (2.4)	−1.2 (2.8)	5.8 (2.4)	3.8 (2.7)	−2.0 (3.0)^‡^	0.8 (−1.3 to 2.8)	0.45
PGIC (*N*=33)^c^		4.6 (1.9)			3.3 (1.6)			0.05
Quality of life total (*N*=34)	27.9 (14.3)	19.6 (17.9)	−8.4 (13.6)^‡^	29.6 (15.7)	24.6 (17.5)	−5.1 (10.5)^a^	−4.0 (−15.1 to 7.1)	0.43
SF McGill Pain Questionnaire Total (*N*=34)	22.6 (11.0)	13.5 (10.0)	−9.1 (11.4)^§^	21.0 (10.0)	20.1 (12.2)	-0.9 (11.3)	−8.2 (−16.1 to −0.3)	0.04
MNSI-Q (*N*=34)	7.5 (2.5)	5.3 (2.5)	−2.2 (2.4)^§^	7.7 (2.8)	7.1 (2.8)	−0.6 (1.5)	−1.6 (−3.0 to −0.2)	0.03
NTSS-6 Total (*N*=34)	10.7 (4.9)	6.8 (4.5)	−3.9 (4.2)^§^	12.3 (5.1)	9.1 (4.0)	−3.2 (3.6)^§^	−0.7 (−3.4 to 2.0)	0.59
BDI (*N*=34)	12.8 (8.1)	8.6 (10.6)	−4.2 (8.8)	11.0 (8.4)	9.5 (7.4)	−1.5 (4.5)	−2.8 (−7.6 to 2.1)	0.26
CESD-R (*N*=34)	11.9 (10.1)	11.2 (13.0)	−0.8 (6.6)	10.8 (7.3)	8.7 (9.2)	−2.1 (7.8)	1.4 (−3.7 to 6.4)	0.59

Abbreviations: BDI, Beck Depression Inventory; BP, blood pressure; CI, confidence interval; CESD-R, Center for Epidemiological Studies, depression scale revised; HDL, high density lipoprotein; LDL, low density lipoprotein; MNSI-PA, Michigan Neuropathy Screening Instrument physical assessment; MNSI-Q, Michigan Neuropathy Screening Instrument questionnaire; NIS-LL, Neuropathy Impairment Score Lower Limb; NTSS-6, Neuropathy Total Symptom Score; PGIC, Patients' Global Impression of Change; Ratio, total cholesterol/HDL; SF, short form; VAS, visual analog pain (worst pain lost two weeks); *Student's *T*-test; ^†^*P*<0.0001; ^§^*P*<0.01; ^‡^*P*<0.05.

a Data are non-normally distributed based on Shapiro-Wilk test.

b Foot and hand conductance in microseimens.

c Since no PGIC measurement was taken at baseline, some statistics were not calculated.
