# Supplementary material for: A dietary intervention for chronic diabetic neuropathy pain: a randomized controlled pilot study
Source: Nutr Diabetes. 2015 May 26;5(5):e158–. doi: 10.1038/nutd.2015.8 (PMC4450462; doi:10.1038/nutd.2015.8)
Supplement: Supplementary Information [file nutd20158x1.doc]

A Dietary Intervention for Chronic Diabetic Neuropathy Pain: A randomized controlled trial

Anne E. Bunner, Cameron Wells, Joseph Gonzales, Ulka Agarwal, Elham Bayat, Neal D. Barnard

Supplemental material

Supplemental Table S1. Intervention effects on clinical measures, completers analysis

|  | Intervention group | | | Supplement group | | | Effect Size | p value* |
| --- | --- | --- | --- | --- | --- | --- | --- | --- |
|  | Baseline | 20 weeks | Change scores | Baseline | 20 weeks | Change scores |  |  |
|  | Mean (SD) | Mean (SD) | Mean (SD) | Mean (SD) | Mean (SD) | Mean (SD) | Mean (95% CI) |  |
| Body weight (kg) (N = 33) | 102.5 (22.7) | 95.5 (20.1) | -7.0 (5.0)† | 103.6 (22.8) | 103.0 (21.9) | -0.6 (3.6) | -6.4 (-9.5 to -3.2) | < 0.001 |
| BMI (N = 33) | 35.9 (6.0) | 33.5 (5.7) | -2.4 (1.5)† | 35.9 (7.2) | 35.7 (7.2) | -0.2 (1.2) | -2.2 (-3.2 to -1.2) | < 0.001 |
| Total cholesterol (N = 33) | 171.2 (48.4) | 159.1 (42.6) | -12.1 (37.8) | 169.0 (38.5) | 171.4 (30.2) | 2.4 (26.4) | -14.5 (-37.8 to 8.8) | 0.21 |
| HDL (N = 33) | 52.1 (15.1) | 46.8 (11.4) | -5.2 (10.1)‡ | 48.2 (19.2) | 45.7 (17.2) | -2.5 (7.1) | -2.7 (-9.0 to 3.5) | 0.38 |
| LDL (N = 32) | 89.0 (38.3) | 81.2 (34.2) | -7.8 (28.9) | 96.0 (36.0) | 95.2 (26.2) | -0.8 (21.8) | -7.0 (-25.6 to 11.7) | 0.45 |
| Ratio (N = 33) | 3.4 (1.1) | 3.5 (0.9) | 0.1 (0.7) | 3.8 (1.2) | 4.1 (1.3) | 0.3 (0.6) | -0.2 (-0.7 to 0.2) | 0.34 |
| Triglycerides (N = 33) | 151.0 (66.3) | 155.7 (61.6) | 4.7 (56.2) | 149.7 (90.8) | 172.9 (114.0) | 23.3 (70.9) | -18.5 (-64.3 to 27.2) | 0.41 |
| Log TG (N = 33) | 2.14 (0.19) | 2.16 (0.19) | 0.01 (0.17) | 2.11 (0.23) | 2.17 (0.25) | 0.06 (0.17) | -0.04 (-0.16 to 0.08) | 0.49 |
| Fasting glucose (N = 33) | 160.6 (73.5) | 134.6 (51.6) | -25.9 (65.0) | 160.3 (61.2) | 139.9 (52.5) | -20.4 (57.2) | -5.6 (-49.1 to 38.0) | 0.80 |
| HbA1c (N = 33) | 8.0 (1.7) | 7.2 (1.4) | -0.8 (1.2)‡ | 7.7 (1.5) | 7.7 (1.4) | -0.1 (1.0) | -0.7 (-1.5 to 0.1) | 0.08 |
| HbA1c, mmol/mL (N = 33) | 64 (19) | 55 (15) | -9 (13)‡ | 61 (16) | 61 (15) | -1 (11) | -8 (-16 to 1) | 0.08 |
| Systolic BP (N = 33) | 139.3 (17.0) | 127.9 (22.6) | -11.5 (22.5) | 144.7 (22.2) | 140.1 (19.9) | -4.5 (15.4) | -6.9 (-20.7 to 6.9) | 0.31 |
| Diastolic BP (N = 33) | 81.5 (9.7) | 75.8 (9.0) | -5.7 (9.4)‡ | 87.2 (15.0) | 84.2 (10.7) | -3.0 (9.1) | -2.7 (-9.3 to 3.9) | 0.41 |
| Foot conductance, avg (N = 19) ¶ | 64.5 (29.5) | 65.2 (24.1) | 0.7 (10.5) | 72.7 (17.7) | 59.7 (24.9) | -13.0 (13.3)‡ | 13.7 (2.2 to 25.2) | 0.02 |
| Hand conductance, avg (N = 19) | 53.2 (28.0) | 55.7 (21.6) | 2.5 (13.9) | 66.3 (24.4) | 59.2 (26.2) | -7.1 (12.2) | 9.6 (-3.1 to 22.3) | 0.13 |
| MNSI-PA (N = 33) | 4.8 (2.4) | 4.3 (2.3) | -0.5 (1.9) | 3.4 (2.2) | 2.6 (1.8) | -0.8 (1.8) | 0.3 (-1.0 to 1.6) | 0.63 |
| NIS-LL (N = 33) | 11.3 (8.0) | 8.7 (6.8) | -2.6 (7.3) | 6.6 (4.8) | 6.8 (5.5) | 0.2 (5.1) | -2.8 (-7.3 to 1.7) | 0.21 |
| VAS (N = 33) | 5.3 (2.7) | 4.0 (2.4) | -1.2 (2.8) | 5.9 (2.5) | 3.7 (2.8) | -2.1 (3.0)‡ | 0.9 (-1.2 to 2.9) | 0.39 |
| PGIC (N = 33)# |  | 4.6 (1.9) |  |  | 3.3 (1.6) |  |  | 0.05 |
| Quality of Life Total (N = 33) | 27.9 (14.3) | 19.6 (17.9) | -8.4 (13.6)‡ | 30.3 (16.0) | 24.9 (18.0) | -5.4 (10.7) | -4.2 (-15.7 to 7.2) | 0.49 |
| SF McGill Pain Questionnaire Total (N = 33) | 22.6 (11.0) | 13.5 (10.0) | -9.1 (11.4)§ | 21.3 (10.3) | 20.4 (12.6) | -0.9 (11.6) | -8.2 (-16.3 to 0.0) | 0.05 |
| MNSI-Q (N = 33) | 7.5 (2.5) | 5.3 (2.5) | -2.2 (2.4)§ | 7.8 (2.9) | 7.2 (2.9) | -0.6 (1.5) | -1.6 (-3.0 to -0.1) | 0.03 |
| NTSS-6 Total (N = 33) | 10.7 (4.9) | 6.8 (4.5) | -3.9 (4.2)§ | 12.4 (5.2) | 9.0 (4.1) | -3.4 (3.6)§ | -0.5 (-3.3 to 2.2) | 0.70 |
| Beck Depression Inventory (N = 33) | 12.8 (8.1) | 8.6 (10.6) | -4.2 (8.8) | 10.6 (8.5) | 9.1 (7.4) | -1.6 (4.7) | -2.7 (-7.7 to 2.4) | 0.28 |
| CESD-R (N = 33) | 11.9 (10.1) | 11.2 (13.0) | -0.8 (6.6) | 10.5 (7.4) | 8.3 (9.3) | -2.3 (8.0) | 1.5 (-3.8 to 6.7) | 0.56 |

* Student’s T-test; †, p < 0.0001; §, p < 0.01; ‡, p < 0.05

¶, Foot and hand conductance in microseimens

#, Since no PGIC measurement was taken at baseline, some statistics were not calculated.

Abbreviations: BMI, Body Mass Index; HDL, high-density lipoprotein cholesterol; LDL, low-density lipoprotein cholesterol; Ratio = Total cholesterol/ HDL; HbA1c, percent hemoglobin A1c; SBP, systolic blood pressure, mmHG; DBP, diastolic blood pressure, mmHG; VAS, visual analog pain scale, worst pain last two weeks; MNSI-PA, Michigan Neuropathy Screening Instrument, physical assessment; NIS-LL, Neuropathy Impairment Score, lower limb; PGIC, Patients’ Global Impression of Change; QOL-T, Norfolk diabetic neuropathy Quality of Life, total score; MPQ-T, Short form McGill Pain Questionnaire total; MNSI-Q, Michigan Neuropathy Screening Instrument, questionnaire; NTSS-6, Neuropathy Total Symptoms Score; BDI, Beck Depression Inventory; CESD-R, Center for Epidemiological Studies, depression scale revised. Cholesterol and triglycerides in mg/dl.

Supplemental Table S2. Short Form McGill Pain Questionnaire Subscores

|  | Intervention group (N = 17) | | | Supplement group (N = 17) | | | Effect Size | p value* |
| --- | --- | --- | --- | --- | --- | --- | --- | --- |
|  | Baseline | 20 weeks | Change scores | Baseline | 20 weeks | Change scores |  |  |
|  | Mean (SE) | Mean (SE) | Mean (SE) | Mean (SE) | Mean (SE) | Mean (SE) | Mean (95% CI) |  |
| Present pain intensity | 2.1 (0.2) | 1.5 (0.3) | -0.6 (0.2)‡ | 2.0 (0.2) | 1.5 (0.3) | -0.5 (0.3) | -0.3 (-1.0 to 0.4) | 0.74 |
| Sensory | 19.6 (2.1) | 11.9 (2.0) | -7.8 (2.1)§ | 18.0 (2.0) | 16.8 (2.3) | -1.2 (2.1) | -6.6 (-12.7 to -0.4) | 0.04 |
| Affective | 3.0 (0.7) | 1.6 (0.5) | -1.4 (0.7) | 3.0 (0.6) | 3.3 (0.8) | 0.3 (0.7) | -1.6 (-3.7 to 0.4) | 0.11 |
| Total | 22.6 (2.7) | 13.5 (2.4) | -9.1 (2.8)§ | 21.0 (2.4) | 20.1 (3.0) | -0.9 (2.7) | -8.2 (-16.1 to -0.3) | 0.04 |

* Student’s T-test; †, p < 0.0001; §, p < 0.01; ‡, p < 0.05

Supplemental Table S3. Norfolk DN Quality of Life Subscores

|  | Intervention group (N = 17) | | | Supplement group (N = 17) | | | Effect Size | p value* |
| --- | --- | --- | --- | --- | --- | --- | --- | --- |
|  | Baseline | 20 weeks | Change scores | Baseline | 20 weeks | Change scores |  |  |
|  | Mean (SE) | Mean (SE) | Mean (SE) | Mean (SE) | Mean (SE) | Mean (SE) | Mean (95% CI) |  |
| Physician | 15.8 (2.0) | 11.5 (3.0) | -4.3 (2.6) | 18.4 (2.6) | 14.9 (2.8) | -3.5 (2.0) | -3.3 (-11.1 to 4.6) | 0.81 |
| Activities of daily life | 2.5 (0.8) | 1.6 (0.6) | -0.9 (0.4) | 2.2 (0.6) | 2.4 (0.8) | 0.2 (0.4) | -0.4 (-2.1 to 1.3) | 0.09 |
| Symptoms | 6.1 (0.5) | 4.3 (0.6) | -1.8 (0.6)§ | 6.7 (0.5) | 5.2 (0.5) | -1.5 (0.5)§ | -0.9 (-2.5 to 0.7) | 0.75 |
| Small fiber | 2.5 (0.9) | 1.6 (0.7) | -0.9 (0.4)‡ | 1.4 (0.5) | 0.9 (0.4) | -0.5 (0.3) | 1.1 (-0.7 to 2.8) | 0.37 |
| Autonomic | 1.1 (0.3) | 0.6 (0.3) | -0.4 (0.2)‡ | 1.1 (0.3) | 1.3 (0.4) | 0.2 (0.3) | -0.4 (-1.2 to 0.4) | 0.05 |
| Total | 27.9 (3.5) | 19.6 (4.3) | -8.4 (3.3)‡ | 29.6 (3.8) | 24.6 (4.2) | -5.1 (2.5) | -4.0 (-15.1 to 7.1) | 0.43 |

* Student’s T-test; †, p < 0.0001; §, p < 0.01; ‡, p < 0.05

Supplemental Table S4. Neuropathic Pain Scale Scores, intention to treat

|  | Intervention group (N = 17) | | | Supplement group (N = 17) | | | Effect Size | p value* |
| --- | --- | --- | --- | --- | --- | --- | --- | --- |
|  | Baseline | 20 weeks | Change scores | Baseline | 20 weeks | Change scores |  |  |
|  | Mean (SE) | Mean (SE) | Mean (SE) | Mean (SE) | Mean (SE) | Mean (SE) | Mean (95% CI) |  |
| Intense pain | 5.6 (0.6) | 3.7 (0.6) | -1.9 (0.6)§ | 5.1 (0.6) | 5.0 (0.6) | -0.1 (0.8) | -1.8 (-3.8 to 0.2) | 0.07 |
| Sharp pain | 5.6 (0.7) | 3.5 (0.5) | -2.1 (0.7)§ | 4.6 (0.8) | 4.1 (0.7) | -0.5 (0.5) | -1.6 (-3.3 to 0.1) | 0.06 |
| Hot pain | 4.8 (0.6) | 2.6 (0.4) | -2.1 (0.5)§ | 4.4 (0.7) | 3.4 (0.6) | -0.9 (0.5) | -1.2 (-2.6 to 0.3) | 0.11 |
| Dull pain | 4.6 (0.6) | 4.4 (0.6) | -0.2 (0.6) | 4.4 (0.6) | 2.8 (0.5) | -1.6 (0.7)‡ | 1.4 (-0.5 to 3.3) | 0.14 |
| Cold pain | 3.9 (0.7) | 2.9 (0.7) | -1.0 (0.5)‡ | 2.8 (0.6) | 2.5 (0.5) | -0.2 (0.5) | -0.8 (-2.1 to 0.6) | 0.26 |
| Skin sensitive to light touch | 4.1 (0.8) | 2.4 (0.5) | -1.6 (0.8)‡ | 2.8 (0.7) | 2.8 (0.7) | -0.1 (0.6) | -1.6 (-3.6 to 0.4) | 0.11 |
| Itchy pain | 2.5 (0.5) | 2.0 (0.5) | -0.5 (0.6) | 3.2 (0.7) | 2.4 (0.6) | -0.8 (0.5) | 0.3 (-1.2 to 1.8) | 0.69 |
| Intolerable pain | 5.4 (0.6) | 3.9 (0.5) | -1.5 (0.6)‡ | 6.2 (0.5) | 3.8 (0.7) | -2.4 (0.6)§ | 0.8 (-1.0 to 2.6) | 0.36 |
| Intensity of deep pain | 5.4 (0.7) | 3.8 (0.7) | -1.6 (0.8) | 5.5 (0.7) | 4.1 (0.7) | -1.4 (0.5)‡ | -0.2 (-2.1 to 1.6) | 0.80 |
| Intensity of surface pain | 4.5 (0.7) | 2.7 (0.4) | -1.8 (0.7)‡ | 4.0 (0.5) | 3.1 (0.6) | -0.9 (0.5) | -0.9 (-2.6 to 0.9) | 0.31 |

* Student’s T-test; †, p < 0.0001; §, p < 0.01; ‡, p < 0.05
